# Proposed recommendations for antithrombotic prophylaxis for children and adolescents with severe infection and/or multisystem inflammatory syndrome caused by SARS-CoV-2

**DOI:** 10.6061/clinics/2020/e2252

**Published:** 2020-11-26

**Authors:** Jorge David Aivazoglou Carneiro, Gabriel Frizzo Ramos, Werther Brunow de Carvalho, Cíntia Johnston, Artur Figueiredo Delgado

**Affiliations:** I Unidade de Hematologia, Departamento de Pediatria, Instituto da Crianca e do Adolescente (ICr), Hospital das Clinicas (HCFMUSP), Faculdade de Medicina, Universidade de Sao Paulo, Sao Paulo, SP, BR.; II Unidade de Terapia Intensiva, Departamento de Pediatria, Instituto da Crianca e do Adolescente (ICr), Hospital das Clinicas (HCFMUSP), Faculdade de Medicina, Universidade de Sao Paulo, Sao Paulo, SP, BR.

Since the end of December 2019, the world has been in the midst of the coronavirus disease (COVID-19) pandemic (1-3). The clinical spectrum of this disease, caused by severe acute respiratory syndrome coronavirus 2 (SARS-CoV-2), ranges from asymptomatic to severe respiratory failure. In pediatric patients, in addition to respiratory symptoms, other findings, such as abdominal pain, diarrhea, vomiting, and skin rash are frequently reported (4-6).

Since March 2020, pediatricians in the United Kingdom and other European countries have observed children hospitalized with SARS-CoV-2 who have subsequently developed severe infection and/or multisystem inflammation. Some of these children have developed shock and multiple organ dysfunction, requiring intensive care, and their disease presentation had some similarities with Kawasaki's disease or toxic shock syndrome (7,8).

Therefore, multisystem inflammatory syndrome in children (MIS-C), temporally associated with infection by SARS-CoV-2, has become a concern among pediatricians in the ongoing worldwide pandemic (9).

We performed a search on PubMed using the keywords “antithrombotic prophylaxis”, “children”, “adolescents”, and “COVID-19”, and did not find any results regarding thromboprophylaxis use in pediatric patients with COVID-19.

Therefore, the aim of the present study was to propose potential recommendations for antithrombotic prophylaxis for children and adolescents with COVID-19, based on the peculiar hemostatic characteristics and thrombotic risk factors for venous thrombosis in children, following general guidelines for antithrombotic therapy in this population (10).

## Hemostasis in children and adolescents: ontogenetic characteristics and impact on pediatric thromboembolism

During phylogenetic evolution, living beings develop a regulatory hemostatic system for protecting the cardiovascular system. In humans, the hemostatic system has a peculiar ontogenesis, which consists of a dynamic evolutionary process deeply influenced by age. The term “developmental hemostasis” has been used since the 1990s to describe age-dependent changes in the hemostatic system, as seen in Table 1 (11). Children are physiologically protected from thromboembolic events due to a decreased capacity for thrombin generation. Thromboses in children are usually related to previously identified risk factors, and only 5% of cases are classified as idiopathic, whereas in adults, this rate reaches 40%. The most frequent risk factors for pediatric venous thromboembolism are catheter use, infections, surgery, trauma, cancer, kidney disease, obesity, and diabetes (12,13).

## Hemostatic changes associated with COVID-19

Infection of the respiratory system and endothelial cells by SARS-CoV-2 causes an intense inflammatory response that includes the activation of the hemostatic system, known as immunothrombosis or thromboinflammation (14,15).

**Figure 1:**
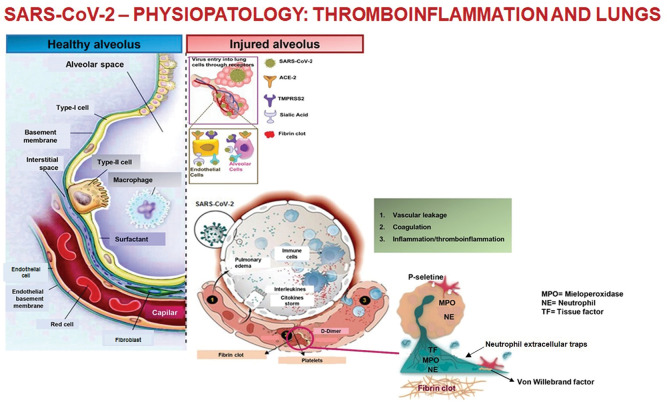
Mechanisms of SARS-CoV-2 Infection on endothelial-lung tissue. Adapted from Pfiefer S, et al. (17) and Teuwen LA, et al. (18).

The inflammatory process involving the vascular alveolar endothelium, even in the initial stages, can trigger the formation of pulmonary clots and neutrophil extracellular traps, which develop to limit infection and viral spread (16,17). These microthrombi may not be easily detected on computed tomography scans because of their small size and because they are limited to the peripheral microvasculature. When not treated, the intense inflammation or cytokine storm can lead to microthrombi expansion, which can manifest clinically as worsening respiratory failure, and radiologically as changes in pulmonary perfusion (Figure 1) (18-19).

Therefore, patients with COVID-19 are prone to develop not only inflammation and hypoxia, but also thromboses, such as pulmonary embolism (20 to 30%), deep venous thrombosis, catheter-associated thrombosis, and arterial thromboses, such as stroke and coronary ischemia. Additionally, microvascular thrombosis, acrosyndrome, and capillary leak, which affect the lungs, kidneys, and heart, add the potential complication of multiple organ dysfunction syndrome (MODS) (20,21).

D-dimer levels have been reported to be the best test for assessing hemostatic changes associated with COVID-19. Several publications have correlated the high concentration of D-dimer with an unfavorable prognosis. Platelet count is usually normal until the patient reached advanced stages of the disease, when mild to moderate thrombocytopenia are observed. Prothrombin time is also normal in most patients, while a significant increase is observed in those who are critically ill. Fibrinogen remains high, except in terminally ill patients, in whom a sharp decrease will occur (22).

Through June 2020, there have been case reports of thrombosis associated with catheter use during MIS-C in pediatric patients. Although there are recommendations for COVID-19 management regarding respiratory support, anti-inflammatory treatment, and antiviral therapy, guidelines regarding thrombotic risk assessment and anticoagulation management of hospitalized children with COVID-19 remain absent (23,24). Additionally, the reported antithrombotic prophylaxis regimens are specific to each tertiary hospital, and are largely based on current adult guidelines (25-27).

## Proposed recommendations for antithrombotic prophylaxis in children and adolescents hospitalized with COVID-19

Our institutional recommendations (ICr-HCFMUSP) (28,29) were adapted from two references (24,30). We recognize that as more children and adolescents with COVID-19 are treated, these recommendations will likely need some modifications.

1) Target population for antithrombotic prophylaxis
  Patients under 18 years of age who are hospitalized with a diagnosis of COVID-19 and present two or more of the following criteria should be considered for prophylactic anticoagulation, after their evaluation by the pediatric hematology staff:
a) Admission to the intensive care unit.
b) Diagnosis of multisystem inflammatory syndrome in children.
c) Risk factors for venous thromboembolism, such as catheter use, immobility, estrogen therapy, malignancy, autoimmune disease, sickle cell disease, obesity, nephrotic syndrome, heart disease, personal or family history of thrombosis, hereditary thrombophilia, and diabetes.
2) Laboratory tests and monitoring
  All hospitalized pediatric patients diagnosed with COVID-19 should undergo the following laboratory tests on admission: blood count with reticulocytes, inflammatory markers (CRP, ESR, IL-6, ferritin), prothrombin time, activated partial thromboplastin time (APTT), fibrinogen, and D-dimer. These values should be monitored regularly, particularly in critically ill patients. The interval between tests should be individualized based on clinical indications and availability of the tests.
3) Antithrombotic prophylaxis for hospitalized pediatric patients
a) Pharmacological antithrombotic prophylaxis is preferred over mechanical prophylaxis.
b) Clinically stable patients who are on prophylactic or therapeutic anticoagulation should maintain anticoagulation at the same dose.
c) Enoxaparin should be initiated in clinically stable patients at the following doses:
  Weight ≤40 kg: 1 mg/kg/dose, once a day
  Weight 40-80 kg: 40 mg/day
d) Enoxaparin 1 mg/kg/dose, twice a day should be initiated in patients at high risk for or already diagnosed thrombosis.
e) Patients who are clinically unstable and/or have renal failure should receive unfractionated heparin at a dose of 10 IU/kg/hour (APTT target 40-70 seconds).
4) Other drugs and pharmacological thromboprophylactic contraindications
a) Do not use oral anticoagulants (warfarin, rivaroxaban, dabigatran, edoxaban, apixaban).
b) Antiplatelet agents are not recommended for venous thromboprophylaxis. They should be used only in cases of Kawasaki disease, according to specific protocols.
c) Contraindications for antithrombotic prophylaxis are shown in Table 2.
5) Duration of pharmacological antithrombotic prophylaxis. Studies in adults have shown that thromboprophylaxis should be maintained throughout hospitalization and for 6-14 days after discharge for patients with conditions that put them at a higher risk of thrombosis, such as malignancy, autoimmune disease, nephrotic syndrome, and immobilization (24,30-33). It is recommended, however, that all pediatric patients should be assessed by the pediatric hematology staff for individualized decisions.

## Figures and Tables

**Table 1:** Coagulation protein values at different ages in relation to those of adults.

	Birth	1 month	6 months	1-5 years	11-16 years
**Pro-coagulants**					
Vitamin K dependent FII FVII FIX FX	lower	lower	lower	lower	lower
FV	lower	lower	lower	lower	lower
FVIII	equal	lower	lower	lower	lower
Contact factors FXI FXII PK HMWK	lower	lower	lower	lower	lower
FXIII	lower	equal	equal	equal	equal
Fibrinogen	equal	equal	equal	equal	equal
Anticoagulants					
**Antithrombin**	lower	lower	lower	lower	lower
α2 Macroglobulin	higher	higher	higher	higher	higher
Protein C	lower	lower	lower	lower	lower
Protein S	lower	lower	lower	equal	equal

Adapted from Jaffray J and Young G (12); Factor II (FII), Factor VII (FVII), Factor IX (FIX), Factor X (FX), Factor V (FV), Factor VIII (FVIII), Factor XI (FXI), Factor XII (FXII), Factor XIII (FXIII), Plasma prekallikrein (PK), High molecular weight kininogen (HMWK).

**Table 2:** Contraindications for pharmacological antithrombotic prophylaxis.

1.	Active bleeding
2.	Acquired bleeding disorders
3.	Anticoagulation
4.	Lumbar puncture or anesthetic procedure expected within the next 12 hours
5.	Lumbar puncture or anesthetic procedure completed less than 4 hours prior
6.	Acute stroke
7.	Thrombocytopenia <25,000/mm^3^
8.	Non-controlled hypertension
9.	Non-treated hereditary hemorrhagic disease

Adapted from Orsi FA et al. (27).
